# Late Presenters with ST-Elevation Myocardial Infarction: A Call to Action

**DOI:** 10.3390/jcm11175169

**Published:** 2022-09-01

**Authors:** Leonardo De Luca, Francesco Antonio Veneziano, Michele Karaboue

**Affiliations:** 1 Department of Cardio-Thoracic-Vascular Sciences, A.O. San Camillo-Forlanini, 00151 Rome, Italy; 2 UniCamillus-Saint Camillus International, University of Health Sciences, 00131 Rome, Italy; 3 Unit of Cardiovascular Science, Department of Medicine, Campus Bio-Medico University, 00128 Rome, Italy; 4 Department of Clinical and Experimental Medicine, University of Foggia, 71121 Foggia, Italy

The emphasis on timely coronary reperfusion in the setting of ST-segment elevation Myocardial Infarction (STEMI) comes from older studies suggesting a significant reduction in mortality among patients treated with fibrinolytic therapy during the first hours after onset of symptoms and a progressive increase in fatal events in those presenting later [1,2,3,4]. In the LATE (Late Assessment of Thrombolytic Efficacy) study, the benefit of fibrinolytic treatment observed in patients presenting early after STEMI onset was not consistent in those enrolled between 12 and 24 h after the onset of chest pain [5]. Later, with the advent and wide usage of percutaneous coronary intervention (PCI) as the preferred method of reperfusion, the question of timing has become more relevant ([6], and international guidelines, based on previous studies on thrombolysis, meta-regression analyses, and in vivo series, recommended coronary revascularization within the first 12 h from symptoms onset [7,8,9].

Pathophysiological studies in animal models suggested the development of irreversible myocardial damage in the first hours after coronary ligation [10,11]. In particular, in mouse models of myocardial infarction with temporary ligation (the so-called “ischemia-reperfusion model”), researchers found that ischemic periods up to 2 h resulted in ischemic-reperfusion infarct sizes of 30%, similarly to the permanent ligation [12,13]. These in vivo studies are in accordance with results from observational studies, which found a direct correlation between survival rates and long-term outcomes when patients were stratified by door-to-balloon time [14,15].

However, unlike what was found in experimental infarction models, a minimal blood flow in the culprit infarcted artery, the possibility of intermittent occlusion, and the presence of collateral vessels could ensure a blood supply sufficient to avoid total necrosis and preserve viable myocardium [16]. To note, rodent and pig models of myocardial infarction are characterized by a nearly complete absence of myocardial collateral circulation [17].

Notably, the overall individual risk profile can widely vary in relation to clinical presentation, cardiovascular risk, and comorbidities. In addition, the occurrence of several chronic ischemic episodes could lead to the development of collateral circulation, which is associated with a better outcome (i.e., lower incidence of cardiogenic shock) [18]. Finally, spontaneous preconditioning and postconditioning may slow the progression of ischemic myocardium to necrosis [19].

A pathological study conducted in 211 patients with STEMI to assess the intracoronary thrombi composition found that in almost half of patients the thrombus presented lytic or organized changes, compatible with an origin of days or weeks before the occlusive event [20]. A similar study showed lytic or organized thrombi in one-third of the cases early after symptom onset (<12 h) and no associations between thrombus composition and epicardial reperfusion grade or the presence of the no-reflow phenomenon [21]. It could therefore be hypothesized that the mechanisms underlying the instability of atherosclerotic plaque occurred several days before symptoms onset, without a strict correlation between them. Therefore, a benefit of mechanical reperfusion could be obtained even behind the recommended 12 h from the symptoms’ onset.

The evidence of myocardial salvage as a result of PCI in late presenters has been investigated in several studies using single-photon emission computed tomography (SPECT) or cardiac magnetic resonance (CMR) imaging. In a study by Schömig et al., 365 asymptomatic STEMI patients, presenting between 12 and 48 h, were randomized to a conservative or an invasive strategy. A significantly reduced infarct size on SPECT was found in the invasive-managed arm, with a not significant trend for a reduction in the secondary combined endpoint of death, recurrent MI, and stroke at 30 days [22]. Accordingly, other studies compared early (<12 h of symptom onset) and late (12–48 h of symptom onset) presenting STEMI patients using CMR. Although these studies documented a larger infarct size and a decreased myocardial salvage in late presenters, the revascularization was still beneficial in these patients, with the possible salvage of the area at risk of >25% [23,24]. 

Recently, in the FAST-MI (French Registry of Acute ST-Elevation or Non-ST-Elevation Myocardial Infarction) registry, 1169 STEMI patients presenting between 12 and 48 h from symptom onset were retrospectively compared with 5104 early comers with STEMI. Late presenters were less likely to receive revascularization. Among PCI-treated patients, compared to those treated medically, all-cause death was significantly lower at 30 days follow-up, with a survival benefit that persisted at 58 months. In multivariate analysis, revascularization of late-comers STEMI patients was independently associated with a significant reduction in mortality occurrence [25]. Accordingly, in an analysis of 910 STEMI patients presenting between 12 and 24 h, invasively treated patients had a significantly lower 12-month mortality compared to those managed conservatively [26].

The overall incidence of late presenters amounts to 15–20% of patients [27,28], and they present with more severe symptoms compared to those who present within 12 h. According to observational studies and registries, these patients are more frequently older and female, with a major prevalence of diabetes mellitus, hypertension, and atypical chest pain at presentation that could be responsible for the delayed hospital admission or correct diagnosis [22,29]. Notably, during the outbreak of SARS-CoV-2, there was an increase in late presenters, as evidenced by some registries [30,31], that led to a significant reduction in the number of pPCIs and an unsurprising increase in AMI complications [32]. The increased likelihood of not undergoing coronary revascularization could contribute to the worse long-term prognosis of late presenters with STEMI [33]. Both in-hospital and up to 3 years of follow-up, the prognosis is worse in proportion to the onset of symptoms [34]. On the other side of the coin, the benefits of revascularization are dubious. Revascularization with PCI in very late presenters (3–28 days) in the OAT (Occluded Artery Trial) study neither led to less mortality rates nor improved left ventricle ejection fraction during 4-year follow-up compared with medical therapy [35]. Similarly, in the DECOPI (DEsobstruction COronaire en Post-Infarctus) study, in which 212 asymptomatic patients with Q-wave on ECG were randomized to PCI or medical therapy, even though invasive treatment was associated with significant improvement in the left ventricular ejection fraction, there was no benefit at 34 months of follow up in terms of cardiac death, non-fatal MI, or ventricular tachyarrhythmia [36]. Therefore, there is clear evidence for a lack of benefit for PCI in STEMI patients admitted to the hospital later than 3 days from symptoms’ onset, but pathophysiological and retrospective observational studies suggest an advantage of coronary revascularization in those presenting from 12 to 72 h from the onset of chest pain (Figure 1).

In this regard, the European Guidelines recommend pPCI in patients presenting with ECG evidence or symptoms consistent with ongoing ischemia, heart failure, shock, or malignant arrhythmias (class I recommendation). In the time window of 12–48 h, routine PCI should be considered in all patients (class IIa). For those presenting beyond 48 h, as resulting from an OAT trial, the ESC discourages routine PCI of the culprit artery (class III), and, similar to chronic occlusion, it is possible to perform either angiography or a non-invasive test for the evaluation of residual myocardial ischemia/viability [7]. The North American Guidelines suggest pPCI between 12 and 24 h of onset only if there is clinical and/or ECG evidence of ongoing ischemia (class IIa) and recommend pPCI for patients with STEMI and cardiogenic shock or acute severe heart failure regardless of the time of onset of symptoms (class I) [8].

In conclusion, STEMI patients with symptoms occurring between 12 and 72 h are increasing and represent a challenge for clinical and interventional cardiologists, with scarce evidence regarding the benefit of revascularization and current pharmacological treatments [22,28]. Therefore, even more than in previous years, there is a need for dedicated prospective studies in order to identify the best management for late presenters with STEMI.

## Figures and Tables

**Figure 1:**
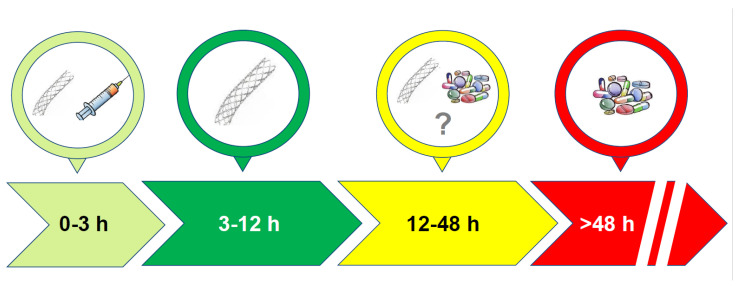
Schematic representation of the evidence on the benefits of coronary revascularization according to patients’ presentation to hospital after the onset of symptoms.

## Data Availability

Not applicable.
